# Association of Infertility Treatment with Perception of Infant Crying, Bonding Impairment and Abusive Behavior towards One’s Infant: A Propensity-Score Matched Analysis

**DOI:** 10.3390/ijerph17176099

**Published:** 2020-08-21

**Authors:** Makiko Sampei, Takeo Fujiwara

**Affiliations:** 1 Department of Global Health Promotion Tokyo Medical and Dental University, Tokyo 113-8519, Japan; sanpei-m@ncchd.go.jp; 2 Department of Social Medicine, National Research Institute for Child Health and Development, Tokyo 157-8535, Japan

**Keywords:** infertility treatment, perception of infant crying, maternal–infant bonding impairment, abusive behavior towards infant, propensity score matching

## Abstract

Background: Although previous qualitative studies suggested the link between infertility treatment and negative emotions towards infants, few empirical population-based studies have investigated the association of infertility treatment with the perception of infant crying, bonding impairment, and abusive behavior towards one’s infant. Methods: Women who participated in a four month health-checkup program in Aichi Prefecture, Japan (*n* = 6590) were asked to a complete a questionnaire that included infertility treatment history, perception of infant crying, maternal–infant bonding impairment assessed by the Mother to Infant Bonding Scale Japanese version, and abusive behavior towards one’s infant. Outcomes were dichotomized, and a conditional logistic regression was applied, using the propensity score match for infertility treatment exposure adjusted for known covariates. Results: A total of 690 participants (11.1%) reported infertility treatment history, and 625 cases were matched. We found that mothers with infertility treatment history were 1.36 times more likely to perceive a higher frequency of infant crying (95% confidence interval (CI):1.05–1.78), but no association with maternal–infant bonding impairment (odds ratio (OR): 1.18; 95% CI: 0.81–1.72) and abusive behavior towards the infant (OR: 0.82; 95% CI: 0.49–1.36). Conclusions: Infertility treatment may be associated with the perception of a higher frequency of infant crying, but it is not associated with bonding impairment and abusive behavior. Further longitudinal study is needed to replicate the findings.

## 1. Introduction

The number of cases of infertility is estimated to be 186 million around the world [1], with Japan having the highest number of infertility treatment cases [2]. The number of births by reproductive technology in Japan in 2017 stood at 56,617; that is, one out of 16 babies born in the country was through infertility treatment [3,4].

Infertility treatment might be a risk factor for child maltreatment (i.e., abusive behavior and neglect), because, in several case studies from Japan, mothers who had undergone infertility treatment were more likely to have negative emotions towards their infant than those who delivered by non-infertility treatment childbirth [5,6,7,8,9,10]. For example, the mother tended to feel her infant as a foreign body, with sentiments such as: “Because my infant has been made artificially, I feel my baby is different from others” or “I feel my body is no longer natural since infertility treatment” [5,6]. Some other case studies reported that mothers who had undergone infertility treatment tended to be ambivalent, with both positive emotions of wanting to love their infant and negative emotions of hating their infant [8,9,10,11,12]. Based on these reports, some researchers proposed special care for mothers who underwent infertility treatment to prevent child maltreatment [5,13,14,15]. At the same time, however, there are previous reviews that reported that mothers who underwent infertility treatment may show higher maternal–fetal attachment during pregnancy [16,17].

To date, few empirical population-based studies have investigated the association of infertility treatment and child maltreatment, especially during infancy. Child maltreatment during infancy includes abusive behaviors, such as shaking and smothering [18,19,20,21,22], and neglect as a result of mother–child bonding impairment, which leads to poor development of attachment for the infant. Furthermore, infant crying is one of the major triggers of infant abuse [18,19,20,21,22] and poor bonding [23,24]. Although infant crying is an attachment behavior; that is, infant crying promotes proximity to the their primary caregiver [25], previous studies shows that infant crying is associated with maternal distress [26], which induces abusive behaviors, such as shaken baby syndrome [20,21,27]. Thus, maternal perception of infant crying needs to be investigated in the context of child maltreatment during infancy. Previous quantitative case-control studies in Australia that investigated the association between infertility treatment and mother–infant bonding at four months did not find any positive association, although the sample size was limited, and sampling was not population-based (*n* = 133) [28,29]. Another study reported no association between infertility treatment history and paternal bonding impairment [30], but the association between infertility treatment history and maternal bonding impairment was not revealed. Thus, there is a need to investigate the association of infertility treatment with perception of infant crying, bonding impairment, and abusive behavior towards one’s infant using a population-based sample.

In this study, we examined the association of infertility treatment with perception of infant crying, bonding impairment, and abusive behavior towards one’s infant, compared to mothers who went through non-infertility treatment childbirth, using a population-based sample in Japan.

## 2. Materials and Methods

### 2.1. Procedure and Participants

This cross-sectional study was conducted in Aichi Prefecture, Japan, between October and November 2012. Aichi Prefecture has 54 municipalities. The population was approximately 7.4 million with 67,913 births in 2012. A total of 45 municipalities, including the prefecture’s capital city Nagoya, participated in this study. The participating municipalities cover 80% of the total population in Aichi. The target subjects were all mothers (*n* = 9707) who were enrolled in a four-month health checkup program between October and November 2012 in participating municipalities. In accordance with the Japanese law (Maternal and Child Health Act), municipalities in Japan provide free infant health checkup at three to four months. Invitations for a health checkup are sent to eligible families. Approximately 96% of Japanese mothers participated in the health checkup [31]. Questionnaires were sent with the invitation by 3 healthcare centers to the target subjects by mail before the start of the health checkup program. Completed questionnaires were collected during the health checkup. Overall, the participation rate for the 3–4 health checkups in the prefecture was 97.9%. In total, 6590 women responded (response rate 68%).

The women who participated were asked to a complete a questionnaire including infertility treatment history of the delivered infant, perception of infant crying, the Mother to Infant Bonding Scale Japanese version (MIBS-J), and self-reported abusive behavior towards infants.

We excluded twin birth samples (*n* = 110; the proportion of the whole sample was 1.7%) as they increase the risk of perception of infant crying [21], mother–infant interaction [32], and abusive behavior [33,34,35], and causes the ambiguity of which out of the infertility treatment and the twin was investigated. We also excluded samples with missing values for infertility treatment history, perception of infant crying, bonding impairment, abusive behavior, and parental age, as parental age is a strong predictor of infertility treatment [36,37] (*n* = 90, 1.4% of the respondent sample). Finally, we analyzed 6213 samples.

### 2.2. Measurements

#### 2.2.1. Infertility Treatment

We assessed infertility treatment history with the following the question: “Did you receive treatment for infertility for this pregnancy?”. The answer selection was “Yes” or “No”. Previous studies showed that self-reported history of infertility treatment by the mother did not differ from that of registered data [38,39].

#### 2.2.2. Perception of Infant Crying

Perception of infant crying was assessed by the statement “My baby cries a lot” using a four-point Likert scale, with 1 indicating “not at all” and 4 indicating “yes, a lot”, based on previous studies [18,19,20,21,22]. Since category 4 represented only a small proportion of the study population, we dichotomized the variable by categorizing responses with 1 and 2 as normal perception of infant crying, and those with 3 and 4 as high perception of infant crying.

#### 2.2.3. Maternal–Infant Bonding Impairment

We evaluated maternal–infant bonding impairment using the 10-item version of the Mother to Infant Bonding Scale Japanese version (MIBS-J) [40] with a validated cut-off point of 4/5 [41]. The self-report scale consists of 10 items including four reverse items on a four-point Likert scale (from 0, “not at all” to 3, “very much”). The total score was calculated for each respondent. The range of the total score was 0–30. High scores denote bonding impairment.

#### 2.2.4. Abusive Behavior towards Infant

We evaluated the two forms of maternal abusive behavior, self-reported shaking and smothering. Parents were asked the frequency of these behaviors over the last month with the following questions: “What is the frequency of shaking your baby violently when he/she cried or expressed frustration?” and “What is the frequency of you smothering your baby with your hands or other objects such as a cushion when he/she cried or expressed frustration?” The response items were “Never”, “1–2 times”, “3–5 times”, “6–10 times”, or “11 or more times”. “No” was assigned to those who responded “Never” while “yes” was assigned to those who responded “1–2 times”, “3–5 times”, “6–10 times”, or “11 or more times”. Furthermore, we assigned these responses as abusive behavior towards infants.

#### 2.2.5. Covariates

We included the following covariates in our model: maternal and paternal age, maternal and paternal employment status, history of maternal depression, maternal and paternal habit during pregnancy (such as smoking), obstetric history (such as abortion), social economic status (such as subjective economic status, number of rooms in the house, and living floor), and living with grandparents. Subsequently, we included the following to control unmeasured variables in our propensity-score matching model: delivery satisfaction, delivery in maternal hometown, infant low birth weight, and feeding status.

### 2.3. Statistical Analysis

First, crude and multiple regressions were performed using the analytic sample (*n* = 6213). Covariates used for the multiple regression were the same 25 variables (See Section 2.2.5) used in propensity-score (PS) matching. Because this study was an observational study, infertility treatment was not randomly assigned. The PS matching method was used to control by matching known covariates simultaneously [42]. PS matching was conducted using the same 25 variables to predict the propensity score for infertility treatment history. The caliper was set as 0.001. Finally, we conducted a conditional logistic regression analysis to compare the outcomes between the matched pairs. Statistical analyses were performed using STATA/SE 15.0 software (Stata Corp Drive, College Station, TX, USA).

### 2.4. Ethics Statement

As the data contained no personal identifiers, the requirement for informed consent was waived. Ethics approval was obtained from the institutional review board of the National Center for Child Health and Development (reference number 611).

## 3. Results

Table 1 shows the demographic characteristics. The average age of mothers and fathers was 31.4 and 33.3 years old, respectively. Fifty percent of the infants were firstborn. A total of 78% of mothers were not working, and 99% were married. Most fathers (98%) were in full-time employment. Almost all mothers did not smoke (97%) or drink (96%) during pregnancy, but about 14% of fathers smoked during the period. Poor economic status made up 11% of the sample.

Table 2 shows the distribution of outcomes. More than 22.8% of mothers perceived their infant to cry a lot (*n* = 1415). The prevalence of bonding impairment with a cut-off score over 5 was 8.6% (*n* = 537), and the prevalence of abusive behavior was 5.3% (*n* = 372).

Table 3 shows the distribution of covariates before and after PS matching by infertility treatment. Among the women who underwent infertility treatment, 83% (*n* = 625) were matched with similar women who went through non-infertility treatment childbirth. The balance of potential confounders within the matched pairs was evaluated using standardized bias. Before PS matching, those who underwent infertility treatment were more likely to be older (34.3 vs. 31.0 years, *p* < 0.01); were in full-time employment (21.2% vs. 15.9%, *p* < 0.01); had a lower prevalence of depression before pregnancy (10.6% vs. 14.8%, *p* < 0.01); had a partner who smoked during the pregnancy period (15.1%, vs. 9.3%, *p* < 0.01); had higher economic status, such as stable economic status (55.9% vs. 43.3%, *p* < 0.01); and had the child as a firstborn (66.7% vs. 48.0%, *p* < 0.01). After PS matching, these variables became nonsignificant, and the bias of these covariates became less than 10%, suggesting that covariates were balanced between the infertility treatment group and the non-infertility treatment group.

We conducted the odds ratio of crude, multiple logistic before PS matching, and conditional logistic regression after PS matching to examine the association between infertility treatment and maternal perception of crying, maternal–infant bonding impairment, and abusive behavior (Table 4). Before PS matching, mothers with infertility treatment showed a higher risk of the perception of a higher frequency of crying in the crude model (odds ratio (OR): 1.24; 95% confidence interval (CI): 1.03–1.48) but no significance was observed in the multiple logistic regression (OR: 1.20; 95% CI: 0.99–1.46). As for bonding impairment and abusive behavior, no association with infertility treatment was observed. After PS matching, mothers with infertility treatment still showed a higher odds ratio of the perception of a higher frequency of crying (OR: 1.36; 95% CI: 1.05–1.78). As for maternal–infant bonding impairment (OR: 1.18; 95% CI: 0.81–1.72) and abusive behavior towards one’s infant (OR: 0.82; 95% CI: 0.49–1.36), no association was found, which was similar to the result before PS matching.

## 4. Discussion

We found that mothers who went through infertility treatment may have the perception of a higher frequency of infant crying, but not bonding impairment and abusive behavior, using propensity-score matching analysis which reduces the bias by unknown variables on the allocation of exposure—in this case, infertility treatment history.

One possible explanation as to why mothers who had infertility treatment history showed a higher risk of the perception of a higher frequency of infant crying might be due to a heightened sensitivity towards their infants. A systematic review reported that mothers who had infertility treatment history were more likely to experience difficulties in parenting [17]. Another study found that mothers who had infertility treatment history were more likely to feel that their infant had a difficult temperament [29]. Thus, the current study adds to the literature that mothers with infertility treatment history may be in more distress due to the perception of a higher frequency of infant crying. Nonetheless, further study is needed to confirm the association between infertility treatment and the actual amount of infant crying. Alternatively, because of the lack of association between infertility treatment and bonding impairment and abusive behavior, the perception of infant crying might be indicative of maternal attentiveness to the infant due to infertility treatment, but not a marker of distress. That is, the response “My baby cries a lot” may not reflect the mother’s attitude towards the infant crying, but the attentiveness to the infant.

To the best of our knowledge, this is the first study to find no association between infertility treatment and bonding impairment and abusive behavior towards one’s infant using a population-based study. Although this is inconsistent with previous Japanese case studies reporting the anecdotal notes from women who underwent infertility treatment and risk of abusive behavior towards infants [5,6,7,8,9,10,11,12,13,14,15,43,44], the findings of the current study are consistent with those of previous quantitative studies [28,29]. A possible reason for this contradiction is that qualitative study is more likely to capture negative emotion towards infants, which might be due to selection bias. If we employ a population-based quantitative study, which has less sampling bias, no association between infertility treatment and bonding impairment is found. Similarly, in this study, mothers who went through infertility treatment showed no bonding impairment and abusive behavior.

This study has several limitations. Because the evaluation of infertility treatment was self-reported, we could not identify the type of infertility treatment (e.g., in vitro fertilization and artificial insemination) and the period of infertility treatment, as well as the differences between them. Abusive behavior was also self-reported. Hence, there was a possibility of underestimating our results. Due to the limited sample location, the results may not be generalized for the whole of Japan. Furthermore, some of the confounding variables, such as maternal educational background and income, were not assessed. Since this is a cross-sectional study, the direction of influence of the relations was unknown.

## 5. Conclusions

In conclusion, infertility treatment may be associated with the perception of a higher frequency of infant crying, but it is not associated with bonding impairment and abusive behavior. To validate the findings, further longitudinal study is needed to investigate the association of infertility treatment with perception of infant crying, maternal–infant bonding impairment, and abusive behavior towards one’s infant.

## Figures and Tables

**Table 1:** Distribution of background characteristics of participants.

Continuing Variables		Participants (*n* = 6213)	
		Mean	SD
Maternal age	-	31.4	4.8
Paternal age	-	33.3	5.6
**Categorical variables**	Response items	*n*	%
Maternal marital status	Married	6145	98.9
	Unmarried	53	0.9
	Missing	15	0.2
Maternal job	Fulltime	1023	16.5
	Part time	299	4.8
	No work	4860	78.2
	Missing	31	0.5
History of depression	No	5311	85.5
	Yes	892	14.4
	Missing	10	0.2
Maternal smoking during pregnancy	No or quit	6025	97.0
	Yes	185	3.0
	Missing	3	0.1
Maternal drinking during pregnancy	No	5961	95.9
	Yes	244	3.9
	Missing	8	0.1
History of abortion	No	5790	93.2
	Yes	419	6.7
	Missing	4	0.1
History of miscarriage	No	5097	82.0
	Yes	1112	17.9
	Missing	4	0.1
History of stillbirth	No	6141	98.8
	Yes	68	1.1
	Missing	4	0.1
Paternal work	Fulltime	6082	97.9
	part time or no-work	106	1.7
	Missing	25	0.4
Paternal smoking during pregnancy	No	5303	85.4
	Yes	897	14.4
	Missing	13	0.2
Talk to partner about raising children	Yes	5814	93.6
	No	174	2.8
	Missing	225	3.6
Subjective economic status	stable	2778	44.7
	afford	2553	41.1
	poor	689	11.1
	Missing	193	3.1
Health insurance	Social insurance	5033	81.0
	National insurance	874	14.1
	Other	16	0.3
	Missing	290	4.7
House type	House	2429	39.1
	Apartment	3740	60.2
	Missing	44	0.7
Living floor	<2F	2099	33.8
	3F–8F	1356	21.8
	>9F	182	2.9
	Missing	2576	41.5
Number of rooms	<2DK	810	13.0
	2LDK-3DK	2090	33.6
	3LDK-4DK	1221	19.7
	>4DK	1877	30.2
	Missing	215	3.5
Live with grandmother	No	5521	88.9
	Yes	692	11.1
Live with grandfather	No	5678	91.4
	Yes	535	8.6
Delivery satisfaction	Satisfaction	5356	86.2
	Slightly satisfied	697	11.2
	Slightly not satisfied	108	1.7
	Not satisfies	34	0.6
	Missing	18	0.3
Delivery in maternal hometown	Yes	2614	42.1
	No	3580	57.6
	Missing	19	0.3
Number of children	Second child or more	3084	49.6
	First child	3112	50.1
	Missing	17	0.3
Feeding at 4 months	Breast-feed	3782	60.9
	Mix-feed	1395	22.5
	Bottle-feed	649	10.5
	Missing	387	6.2
Low birth weight	<2500	454	7.3
	≥2500	5736	92.3
	Missing	23	0.4

**Table 2:** Distribution of outcomes of participants.

Variables	Response Items	Participants (*n* = 6213)	
		N ^a^	%
Perception of infant crying	No	4798	77.2
	Yes	1415	22.8
Bonding impairment	No	5676	91.4
	Yes	537	8.6
Abusive behavior towards infant	No	5881	94.7
	Yes	332	5.3

^a^ Number of observations.

**Table 3:** Distribution of characteristic covariates for the history of infertility treatment before and after propensity score (PS) matching (outcome is “Maternal perception of crying”).

Variables	Response Items	*n* = 6213				*n* = 1250			
		Before PS Matching				After PS Matching			
		History of Infertility Treatment		*p* ^a^	Bias (%)	History of Infertility Treatment		*p* ^a^	Bias (%)
Variables		(Number of samples, proportions)				(Number of samples, proportions)			
		No	Yes			No	Yes		
		(5523, 88.9%)	(690, 11.1%)			(*n* = 625)	(*n* = 625)		
		Mean (SD)				Mean (SD)			
Maternal age		31.0 (4.7)	34.3 (4.3)	<0.01	73.5	33.9 (4.4)	33.8 (4.2)	0.9	−0.6
Paternal age		33.0 (5.5)	36.0 (5.1)	<0.01	57.4	35.5 (5.5)	35.6 (4.9)	0.8	1.7
		*n* (%)				*n* (%)			
Maternal marital status	Unmarried	51 (0.9)	2 (0.3)	0.2	Reference	3 (0.5)	2 (0.3)	0.8	Reference
	Married	5459 (98.8)	686 (99.4)		−8.2	621 (99.4)	621 (99.4)		−2.1
	Missing	13 (0.2)	2 (0.3)		1.1	1 (0.2)	2 (0.3)		3.1
Maternal job	Fulltime	877 (15.9)	146 (21.2)	<0.01	Reference	135 (21.6)	127 (20.3)	0.3	Reference
	Part timer	263 (4.8)	36 (5.2)		2.1	37 (5.9)	31 (5.0)		−4.4
	No work	4355 (78.9)	505 (73.2)		−13.3	453 (72.5)	464 (74.2)		4.1
	Missing	28 (0.5)	3 (0.4)		−1.1	0 (0)	3 (0.5)		7.0
History of depression	No	4695 (85.0)	616 (89.3)	0.01	Reference	559 (89.4)	556 (89.0)	0.8	Reference
	Yes	819 (14.8)	73 (10.6)		−12.8	64 (10.2)	68 (10.9)		1.9
	Missing	9 (0.2)	1 (0.1)		−0.5	2 (0.3)	1 (0.2)		−4.1
Maternal smoking during pregnancy	No or quit	5345 (96.8)	680 (98.6)	0.04	Reference	608 (97.3)	616 (98.6)	0.1	Reference
	Yes	175 (3.2)	10 (1.5)		−11.5	17 (2.7)	9 (1.4)		−8.5
	Missing	3 (0.1)	0 (0)		−3.3				0.0
Maternal drinking during pregnancy	Yes	216 (3.9)	28 (4.1)	0.6	Reference	29 (4.6)	24 (3.8)	0.5	Reference
	No	5299 (95.9)	662 (95.9)		0.0	596 (95.4)	601 (96.2)		4.1
	Missing	8 (0.1)	0 (0)		−5.4				0.0
History of abortion	No	5127 (92.8)	663 (96.1)	<0.01	Reference	600 (96.0)	601 (96.2)		Reference
	Yes	392 (7.1)	27 (3.9)		−14.0	25 (4.0)	24 (3.8)		−0.7
	Missing	4 (0.1)	0 (0)		−3.8				0.0
History of miscarriage	No	4594 (83.2)	503 (72.9)	<0.01	Reference	465 (74.4)	467 (74.7)		Reference
	Yes	925 (16.8)	187 (27.1)		25.2	160 (25.6)	158 (25.3)		−0.8
	Missing	4 (0.1)	0 (0)		−3.8				0.0
History of stillbirth	No	5461 (98.9)	680 (98.6)	0.5	Reference	618 (98.9)	615 (98.4)	0.5	Reference
	Yes	58 (1.1)	10 (1.5)		3.6	7 (1.1)	10 (1.6)		4.3
	Missing	4 (0.1)	0 (0)		−3.8				0.0
Paternal work	Fulltime	5400 (97.8)	682 (98.8)	0.2	Reference	619 (98.7)	617 (98.9)	0.8	Reference
	part time or no-work	100 (1.8)	6 (0.9)		−8.2	4 (0.6)	6 (1.0)		2.8
	Missing	23 (0.4)	2 (0.3)		−2.1	2 (0.3)	2 (0.3)		0.0
Paternal smoking during pregnancy	Yes	833 (15.1)	64 (9.3)	<0.01	Reference	57 (9.1)	59 (9.4)	0.6	Reference
	No	4678 (84.7)	625 (90.6)		17.9	568 (90.9)	565 (90.4)		−1.5
	Missing	12 (0.2)	1 (0.1)		−1.7	0 (0)	1 (0.2)		3.8
Talk to partner about raising children	Yes	5156 (93.4)	658 (95.4)	0.1	Reference	586 (93.8)	595 (95.2)	0.4	Reference
	No	160 (2.9)	14 (2.0)		−5.6	14 (2.2)	13 (2.1)		−1.0
	Missing	207 (3.8)	18 (2.6)		−6.5	25 (4.0)	17 (2.7)		−7.3
Subjective economic status	stable	2392 (43.3)	386 (55.9)	<0.01	Reference	321 (51.4)	341 (54.6)	0.6	Reference
	afford	2308 (41.8)	245 (35.5)		−12.9	235 (37.6)	227 (36.3)		−2.6
	poor	645 (11.7)	44 (6.4)		−18.6	50 (8.0)	43 (6.9)		−3.9
	Missing	178 (3.2)	15 (2.2)		−6.5	19 (3.0)	14 (2.2)		−4.9
Health insurance	Social insurance	4446 (80.5)	587 (85.1)	0.02	Reference	514 (82.2)	528 (84.5)	0.6	Reference
	National insurance	803 (14.5)	71 (10.3)		−12.9	76 (12.2)	67 (10.7)		−4.4
	Other	15 (0.3)	1 (0.1)		−2.8	3 (0.5)	1 (0.2)		−7.0
	Missing	259 (4.7)	31 (4.5)		−0.9	32 (5.1)	29 (4.6)		−2.3
House type	House	2116 (38.3)	313 (45.4)	<0.01	Reference	281 (45.0)	281 (45.0)	0.9	Reference
	Apartment	3366 (61.0)	374 (54.2)		−13.7	340 (54.4)	341 (54.6)		0.3
	Missing	41 (0.7)	3 (0.4)		−4.0	4 (0.6)	3 (0.5)		−2.1
Living floor	<2F	1911 (34.6)	188 (27.3)	<0.01	Reference	181 (29.0)	177 (28.3)	0.8	Reference
	3F–8F	1209 (21.9)	147 (21.3)		−1.4	134 (21.4)	133 (21.3)		−0.4
	>9F	150 (2.7)	32 (4.6)		10.2	18 (2.9)	24 (3.8)		5.1
	Missing	2253 (40.8)	323 (46.8)		12.1	292 (46.7)	291 (46.6)		−0.3
Number of rooms	<2DK	760 (13.8)	50 (7.3)	<0.01	Reference	42 (6.7)	48 (7.7)	0.8	Reference
	2LDK-3DK	1885 (34.1)	205 (29.7)		−9.5	205 (32.8)	192 (30.7)		−4.5
	3LDK-4DK	1052 (19.1)	169 (24.5)		13.2	143 (22.9)	148 (23.7)		1.9
	>4DK	1629 (29.5)	248 (35.9)		13.8	221 (35.4)	219 (35.0)		−0.7
	Missing	197 (3.6)	18 (2.6)		−5.5	14 (2.2)	18 (2.9)		3.7
Live with grandmother	No	4913 (89.0)	608 (88.1)	0.5	Reference	538 (86.1)	552 (88.3)	0.2	Reference
	Yes	610 (11.0)	82 (11.9)		2.6	87 (13.9)	73 (11.7)		−7.0
Live with grandfather	No	5047 (91.4)	631 (91.5)	1.0	Reference	564 (90.2)	573 (91.7)	0.4	Reference
	Yes	476 (8.6)	59 (8.6)		−0.2	61 (9.8)	52 (8.3)		−5.1
Delivery satisfaction	Satisfaction	4788 (86.7)	568 (82.3)	0.02	Reference	511 (81.8)	519 (83.0)	0.7	Reference
	Slightly satisfied	595 (10.8)	102 (14.8)		12.0	100 (16.0)	88 (14.1)		−5.8
	Slightly not satisfied	97 (1.8)	11 (1.6)		−1.3	7 (1.1)	11 (1.8)		5.0
	Not satisfied	28 (0.5)	6 (0.9)		4.4	5 (0.8)	4 (0.6)		−1.9
	Missing	15 (0.3)	3 (0.4)		2.7	2 (0.3)	3 (0.5)		2.7
Delivery in maternal hometown	Yes	2326 (42.1)	288 (41.7)	0.4	Reference	258 (41.3)	254 (40.6)	0.9	Reference
	No	3182 (57.6)	398 (57.7)		0.1	363 (58.1)	368 (58.9)		1.6
	Missing	15 (0.3)	4 (0.6)		4.7	4 (0.6)	3 (0.5)		−2.5
Number of children	Second child or more	2857 (51.7)	227 (32.9)	<0.01	Reference	224 (35.8)	224 (35.8)	0.9	Reference
	First child	2652 (48.0)	460 (66.7)		38.4	398 (63.7)	399 (63.8)		0.3
	Missing	14 (0.3)	3 (0.4)		3.1	3 (0.5)	2 (0.3)		−2.7
Feeding at 4 months	Breast-feed	3396 (61.5)	386 (55.9)	<0.01	Reference	359 (57.4)	362 (57.9)	0.9	Reference
	Mix-feed	1200 (21.7)	195 (28.3)		15.1	162 (25.9)	169 (27.0)		2.6
	Bottle-feed	583 (10.6)	66 (9.6)		−3.3	63 (10.1)	59 (9.4)		−2.1
	Missing	344 (6.2)	43 (6.2)		0.0	41 (6.6)	35 (5.6)		−4.0
Low birth weight	≥2500	5112 (92.6)	624 (90.4)		Reference	563 (90.1)	569 (91.0)		Reference
	<2500	392 (7.1)	62 (9.0)	0.1	6.9	58 (9.3)	52 (8.3)	0.8	−3.5
	Missing	19 (0.3)	4 (0.6)		3.5	4 (0.6)	4 (0.6)		0.0

^a^*p*-value for that continuous variables were calculated using a t-test, categorical variables were calculated using a chi-square test.

**Table 4:** Relationship between the history of infertility treatment and outcomes (perception of infant crying, bonding impairment, and abusive behavior) for before and after propensity score (PS) matching.

		Perception of infant crying					
		OR ^b^ (95% CI) ^c^					
		Crude analysis		Multiple logistic regression		After PS matching	
		N ^d^ = 6213	*p* ^a^	N ^d^ = 6213	*p* ^a^	N ^d^ = 1250	*p* ^a^
History of infertility treatment	Yes	1.24 (1.03–1.48)	0.02	1.20 (0.99–1.46)	0.06	1.36 (1.05–1.78)	0.02
	No	Reference		Reference		Reference	
		**Bonding impairment**					
		**OR ** ^**b**^ ** (95% CI) ** ^**c**^					
		Crude analysis	*p* ^a^	Multiple logistic regression	*p* ^a^	After PS matching	*p* ^a^
History of infertility treatment	Yes	1.22 (0.94–1.59)	0.14	1.11 (0.83–1.48)	0.48	1.18 (0.81–1.72)	0.39
	No	Reference		Reference		Reference	
		**Abusive behavior**					
		**OR ** ^**b**^ ** (95% CI) ** ^**c**^					
		Crude analysis	*p* ^a^	Multiple logistic regression	*p* ^a^	After PS matching	*p* ^a^
History of infertility treatment	Yes	0.76 (0.51–1.12)	0.16	0.91 (0.60–1.38)	0.66	0.82 (0.49–1.36)	0.44
	No	Reference		Reference		Reference	

^a^*p*-value; ^b^ odds ratio; ^c^ confidence interval; ^d^ number of observations.
